# A Small Vimentin-Binding Molecule Blocks Cancer Exosome Release and Reduces Cancer Cell Mobility

**DOI:** 10.3389/fphar.2021.627394

**Published:** 2021-07-08

**Authors:** Jianping Wu, Qian Xie, Yanjun Liu, Yanan Gao, Zhipeng Qu, Lian Mo, Ying Xu, Ruihuan Chen, Liyun Shi

**Affiliations:** ^1^ School of Medicine & Holistic Integrative Medicine, Nanjing University of Chinese Medicine, Nanjing, China; ^2^ Luoda Biosciences, Inc., Chuzhou, China; ^3^ Cambridge-Suda Genomic Resource Center, Medical College of Soochow University, Suzhou, China; ^4^ Aluda Pharmaceuticals, Inc., Menlo Park, CA, United States

**Keywords:** small molecule, vimentin, exosomes, inhibitor, cancer mobility

## Abstract

Vimentin is an intermediate filament protein with diverse roles in health and disease far beyond its structural functions. Exosomes or small extracellular vesicles (sEVs) are key mediators for intercellular communication, contributing to tissue homeostasis and the progression of various diseases, especially the metastasis of cancers. In this study, we evaluated a novel vimentin-binding compound (R491) for its anti-cancer activities and its roles in cancer exosome release. The compound R491 induced a rapid and reversible intracellular vacuolization in various types of cancer cells. This phenotype did not result in an inhibition of cancer cell growth, which was consistent with our finding from a protein array that R491 did not reduce levels of major oncoproteins in cancer cells. Morphological and quantitative analyses on the intracellular vacuoles and extracellular exosomes revealed that in response to R491 treatment, the exosomes released from the cells were significantly reduced, while the exosomes retained as intra-luminal vesicles inside the cells were subsequently degraded. Vim^+/−^ cells had lower amounts of vimentin and accordingly, lower amounts of both the retained and the released exosomes than Vim^+/+^ cells had, while the vimentin-binding compound R491 inhibited only the release of exosomes. Further functional tests showed that R491 significantly reduced the migration and invasion of cancer cells *in vitro* and decreased the amount of exosome in the blood in mice. Our study suggests that vimentin promotes exosome release, and small-molecule compounds that target vimentin are able to both block cancer exosome release and reduce cancer cell motility, and therefore could have potential applications for inhibiting cancer invasive growth.

## Introduction

Exosomes are small extracellular vesicles (sEVs) formed by inward budding of the late endosomal membrane within multivesicular bodies (MVB) and are released via the fusion of MVBs with the cell membrane. These vesicles carry a variety of biologically active molecules and transfer their contents between cells locally and systemically, thereby facilitating normal physiological processes and exacerbating pathological alterations (van Niel et al., 2018; Pegtel and Gould, 2019; Kalluri and LeBleu, 2020). Exosomes have been extensively investigated for clinical applications as diagnostic markers (Shah et al., 2018; Xu et al., 2018; Jalalian et al., 2019), as drug delivery vehicles (Kamerkar et al., 2017; Fu et al., 2019; Yang et al., 2020) and directly as therapeutic agents (Saha et al., 2019; Zhang et al., 2019; Zipkin, 2019; Dinh et al., 2020). Modulating exosome release appears to be an attractive therapeutic intervention (McAndrews and Kalluri, 2019), especially in immuno-oncology because of the critical roles of exosomes in tumor progression and immune evasion (Hoshino et al., 2015; Chen et al., 2018; Poggio et al., 2019; Wortzel et al., 2019; Daassi et al., 2020). Despite various drug discovery attempts from empirical study to drug repurposing and high throughput screening (Trajkovic et al., 2008; Chalmin et al., 2010; Ostrowski et al., 2010; Datta et al., 2018; Kosgodage et al., 2018; Im et al., 2019; Kulshreshtha et al., 2019), targeting exosomes for the treatment of disease has been challenging due to exosome’s complex biogenesis, dynamic contents, heterogeneous origins, and diverse functions (Jeppesen et al., 2019; Pegtel and Gould, 2019; Kalluri and LeBleu, 2020).

Vimentin is an intermediate filament protein expressed abundantly in cells of mesenchymal origin, and also dynamically in various types of cells during different differentiation stages. As a component of the cytoskeleton, it not only confers mechanical resistance to cells but also serves as a signaling scaffold for a number of proteins involved in attachment, migration, invasion and differentiation, etc. (Danielsson et al., 2018). Vimentin associates with key proteins of the vesicular membrane transport machinery, helping to provide tracks for vesicle transport, position endosomes and lysosomes, maintain the integrity of various organelles, drive the exosome-mediated epithelial to mesenchymal transition (Styers et al., 2006; Margiotta and Bucci, 2016; Rahman et al., 2016). We reasoned that targeting vimentin might interfere with vesicle transport and thereby modulate exosome release. To prove this hypothesis, we developed a series of small molecule compounds that specifically bind to vimentin (Zhang et al., 2021).

In the current study we evaluated the biological activities of a novel vimentin-targeting small molecule compound (R491) in cell lines of human lung, brain, gastric, liver, and pancreatic cancers, and investigated their underlying mechanisms.

## Materials and Methods

### Drug Preparation

The vimentin-binding compound used in this study was R491, (E) - 1 - (4 - fluorophenyl) - 3 - (4 - (4 - (morpholine - 1 - yl) - 6 - styryl - 1, 3, 5 - triazinyl - 2 - amino) phenyl) urea. It has a molecular formula of C28H26FN7O2 and a molecular weight of 511.55. The compound was synthesized at Bellen Chemistry Co., Ltd., analyzed at Porton Pharma Solutions, Ltd., and provided by Luoda Biosciences, Inc. The compound had a ≥ 98% purity (Certificate of Analysis in Supplementary Table S1). The compound was dissolved in DMSO (Solarbio, Beijing, China) for *in vitro* experiments. The stock solution of 20 mM was stored at -20°C and diluted with culture media to working concentrations prior to use.

### Cell Culture

Human non-small cell lung cancer A549 cells, human pancreatic cancer PANC-1 cells, human glioma U87 cells, human hepatoma cancer HUH7 cells and SMMC-7721 cells, and human gastric cancer AGS cells were purchased from ATCC or Stem Cell Bank, Chinese Academy of Sciences (Shanghai, China). The cells were maintained at 37°C in RPMI1640 medium (Gibco, United States) containing 10% fetal bovine serum (Sigma, United States), 100 U penicillin and 100 μg/ml streptomycin (Gibco, United States) in a humidified atmosphere containing 5% CO_2_.

### Morphological Analysis

The cells were seeded in 96-well plates at 3 × 10^3^ cells per well and cultured at 37°C for 24 h. Then, the cells were treated with DMSO and the compound R491 of different concentrations respectively and cultured for different durations. The effects of R491 on cell morphology were examined under a phase-contrast microscope.

### Cell Proliferation Assay

Cell proliferation was measured by using a colorimetric assay kit, CCK-8 (Dojindo, Japan). Briefly, cells in the exponential phase of growth were harvested; 3 × 10^3^ single cells were seeded in each well of 96-well plates and cultured overnight. The cells in each well were added with either DMSO as vehicle control or the compound R491 at different concentrations and cultured for 72 h. Then, 10 μl of the CCK-8 solution was added to each well of the culture and the plates were incubated at 37°C for 1–2 h. The absorbance at 450 nm was measured using a multi-function microplate reader (PerkinElmer, EnSpire, America). The cell count was calculated based on the absorbance. Triplicates were run in each of three independent tests.

### Protein Chip Analysis

Lysates from A549 and PANC-1 cells treated with 3 μM of R491 or equal volume of DMSO were extracted with Lysis Buffer 17 (R and D systems, Abingdon, United Kingdom) supplemented with 10 μg/ml Aprotinin (Sigma, Shanghai, China), 10 μg/ml Leupeptin (Sigma, Shanghai, China), and 10 μg/ml Pepstatin (Sigma, Shanghai, China). A total of 200 μg protein of the lysate was run on each array of the Human XL Oncology Array Kit (R and D systems, Abingdon, United Kingdom). The procedures were followed strictly in accordance with the manufacturer’s instructions. The membrane chips were wrapped in plastic wraps and exposed to X-ray films for 8 min. The optical densities on the developed X-ray film (hu.q, HQ-320XT, China) were quantified by a transmission-mode scanner (EPSON, Beijing, China) and analyzed by Image J software.

### Transmission Electron Microscopy and MVB Quantification

Cells were harvested and fixed overnight at 4°C in 0.1 M sodium phosphate buffer containing 2.5% glutaraldehyde. Then, the cell pellets were washed in phosphate buffer and incubated with 1% OsO_4_ for 90 min at 4°C. Samples were dehydrated, embedded in Spurr’s resin and sectioned. Ultrathin sections (50–70 nm) were then fixed with 2% uranyl acetate for 10 min and with a lead-staining solution for 5 min. A Tecnai G2 Spirit Bio TWIN transmission electron microscope (FEI, Hillsboro, United States) was used to analyze the intracellular morphological changes. MVBs, atypical MVB Ⅱ and empty vacuoles inside the cells were identified and counted based on their morphological characteristics. A minimum of 20 MVBs in each of at least 20 cells from each experiment were analyzed for quantifying intraluminal vesicles (ILVs). Data were collected from two independent experiments.

The morphology of exosomes prepared from culture supernatants was examined by transmission electron microscopy (TEM). The exosome pellets were fixed in 2% glutaraldehyde overnight at 4°C. A drop of 10 μL exosome suspension was loaded onto Formvar/Carbon–coated TEM copper grids and allowed to stand for 20 min. Excess fluid was drawn off. The sample was post-fixed with 1% uranyl acetate for 5 min, washed with double distilled water for 8 times, and allowed to dry under an electric incandescent lamp for 10 min before being examined with a Tecnai G2 Spirit Bio TWIN transmission electron microscope (FEI, Hillsboro, United States) operated at 120 kV.

### Immunofluorescence Microscopy

To detect CD63, cells were seeded in the wells of a 24-well plate, which contained a circular slide at the bottom of each well and were cultured overnight. Different concentrations of R491 were then added into the wells and the cells were cultured for 48 h. The cells were washed with phosphate buffer three times, fixed with 4% paraformaldehyde, and then stained with the anti-CD63 antibody (1: 300, Abcam, United Kingdom) followed by the secondary antibody (1: 1,000). After the cells were washed with phosphate buffer three times, the cells were then stained by DAPI (1: 1,000). The slides were examined using a Leica SP5 confocal microscope (Leica, GER). The images were processed and assembled using Leica software and quantified using Image J software.

### Small Extracellular Vesicle (sEV)/Exosomes Purification, Characterization and Analysis

To purify sEVs or exosomes from cell culture supernatants, the cells were cultured for 3 days in DMEM media supplemented with 10% exosome-free FBS, which was prepared from regular FBS by ultracentrifugation at 120,000 g for 16 h. The conditioned culture media were collected and centrifuged at 500 g for 5 min and then 2,000 g for 20 min to remove dead cells and cell debris. Then, large vesicles were removed through centrifugation at 12,000 g for 30 min. Crude sEVs were pelleted via ultracentrifugation of the supernatant at 110,000 g for 70 min (Backman Ti70), washing with PBS and filtered (0.2 µm). Then the pellets were suspended and ultra-centrifugated again at 110,000 g for 70 min. All ultracentrifugation was performed at 4°C. The size and number of sEVs were analyzed using the LM10 nanoparticle characterization system (NanoSight) equipped with a blue laser (405 nm).

### *In Vivo* Studies

The BALB/c mice (18∼20 g) aged 6∼8 weeks were purchased from Beijing Vital River Laboratory Animal Technology Co., Ltd. (Beijing, China) and housed in the Laboratory Animal Center of Nanjing University of Chinese Medicine. Animals were maintained at 22 ± 2°C on a regular light-dark cycle with free access to food and water. All animal experiments were approved by the Nanjing University of Chinese Medicine ethics committee. Animal care was provided in accordance with guidelines approved by the IACUC.

Three mice per group were given 100 mg/kg of R491 or the same volume of vehicle once per day for 14 consecutive days. 24 h after the last administration, the blood samples were taken from the mice under anesthesia with pentobarbital sodium, then the mice were euthanized. Plasma exosomes were prepared by sucrose density gradient centrifugation and analyzed by Western blot.

### Isolation of sEVs From Mouse Plasma

To obtain plasma for sEV isolation, blood was centrifuged at 4°C at 500 × g for 5 min; 2000 × g for 15 min; and 10,000 × g for 20 min to remove cells, debris and large vesicles. The sEV pellet was resuspended in 60 ml of cold PBS, and the sEV suspension was centrifuged at 100,000 × g for 70 min at 4°C. For sucrose density gradient centrifugation, the washed sEV pellet was mixed with 2 ml of a 2.5 M solution of sucrose solution and inserted inside a sucrose step gradient column (ten 2-ml steps starting from 2 to 0.4 M sucrose using 20 mM HEPES as the diluents). The sucrose step gradient was centrifuged at 200,000 × g (Backman Ti70) for 16 h at 4°C. The fractions were collected and centrifuged in cold PBS at 100,000 × g for 70 min at 4°C. All the fraction pellets were resuspended in 30 μl lysis buffer before further western blot analysis.

### CRISPR Design and Vimentin Gene Editing

The plasmid PB-TRE-NLS-linker-Cas9-ZF-IRES-hrGFP-Blasticidin was used for the doxycycline-inducible expression of human codon-optimized Cas9, and the plasmid pGL3-U6-2sgRNA-ccdB-EF1a-Puromycin (Gifts from Dr. Xingxu Huang), for the expression of sgRNA specific to vimentin gene. With the aid of the CRISPR Design tool (http://crispr.mit.edu/), two guide strands were designed to target two segments in the Exon1 of human vimentin gene (target site 1: GTC​CTC​GTC​CTC​CTA​CCG​C; target site 2: CGG​CTC​CTG​CAG​GAC​TCG​G). The sgRNA-coding sequences were cloned into the sgRNA-expressing vector at BsmBI site. U87 cells were transfected with the Cas9-expressing plasmid by Lipofectamine 2000 (Invitrogen) and selected by blasticidin. Stable lines of the cells were treated with doxycycline for 12 h to induce Cas9 expression, then transfected with the vimentin sgRNA-expressing vector, and after 24 h, selected by puromycin for transduced cells. The pooled and clonal cells were collected. The deletion in the targeted region of vimentin gene and the absence of vimentin protein were confirmed by PCR and Western blotting, respectively. The PCR primes were forward primer ATG​TTC​GGC​GGC​CCG​GGC​AC and reverse primer AGG​AGC​CGC​ACC​CCG​GGC​ACG for knockout genotype, and forward primer GAG​GGG​ACC​CTC​TTT​CCT​AA and reverse primer GGT​GGA​CGT​AGT​CAC​GTA​GC for wildtype genotype.

### Western Blot

sEVs were purified from different conditioned cultures using a standard differential centrifugation protocol, then lyzed by RIPA buffer containing PMSF (Beyotime Biotechnology, Shanghai, China). Cells were directly lyzed by RIPA/PMSF buffer. Both of the lysates were cleared by centrifugation to remove insoluble. The protein concentrations were measured by an Enhanced BCA Protein Assay Kit (Beyotime Biotechnology, Shanghai, China). 20 μg of the exosomal proteins or 40 μg of the cellular proteins were separated by sodium dodecyl sulfate-polyacrylamide gel electrophoresis (SDS-PAGE) and transferred onto polyvinylidene fluoride membrane (Merck Millipore). The membranes were blocked by 5% milk for 1 h, and then incubated with primary antibody at 4°C overnight, washed three times, then incubated with secondary antibody at room temperature for 1 h. The protein bands were detected by Gel Imaging System (Bio-Rad, United States), and measured by Image J. The primary antibodies used were rabbit anti-GAPHD, *β*-actin, Vimentin, CD63, CD9, and TSG101 (Abcam, United Kingdom).

### Wounding Healing Assay

The cells were seeded in 6-well plates and cultured in complete medium at 37°C in an atmosphere containing 5% CO_2_. Once the cells reached 90% confluence, a plastic 200 µL pipette tip was used to create vertical wounds. Then, washed the cells three times with PBS and subsequently incubated with serum-free RPMI-1640 medium containing different concentrations of R491 for 24 h. Phase-contrast microscopy was employed to photograph the wounded field and migrated cells within the wounded region. The wounded area was measured using Image J software.

### Invasion Assay

1 × 10^4^ cells in suspended in 100 µL serum-free RPMI-1640 medium were seeded in each upper chamber of a 6-well transwell (Cat. 3,422. Corning, NY, United States) that was precoated with Matrigel, added with DMSO or R491 at different concentrations. 600 µL RPMI-1640 with 10% FBS was added to the bottom chamber of each well. The cells in the transwell plate were cultured for 24 h. The upper surface cells were gently wiped by a moistened cotton swab, while the cells that traversed the Matrigel to the lower surface of membrane were fixed with paraformaldehyde and stained with 0.1% crystal violet. These cells on the lower surface were considered invasive and were counted in five random fields under an inverted microscope. The number of invasive cells was calculated by Image J software.

### Statistical Analysis

The statistical analysis was performed using GraphPad Prism 6.0. Data were presented as individual value or mean ± SEM. Differences between groups were analyzed by Student’s t test or Mann–Whitney test or Fisher’s exact test. *p* < 0.05 was considered statistically significant.

## Results

### The Vimentin-Binding Compound R491 Induced a Strong, Rapid and Reversible Intracellular Vacuolization in Multiple Types of Cancer Cells

**FIGURE 1:**
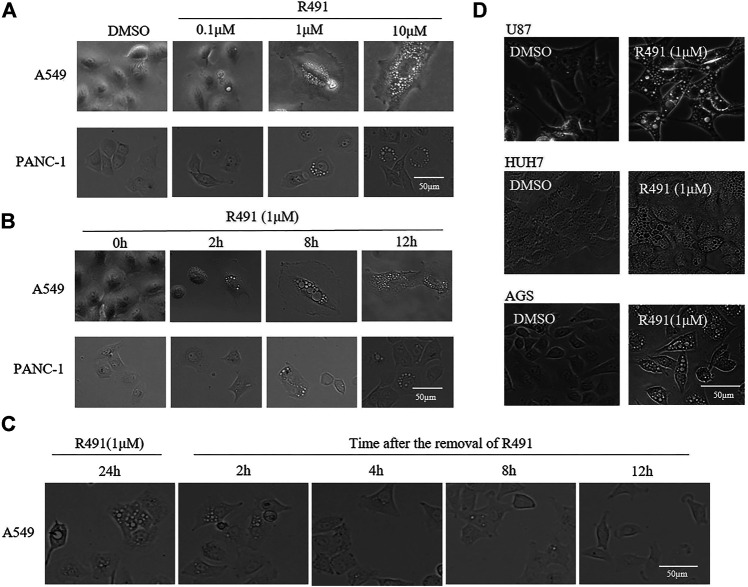
The vimentin-binding compound R491 induced a rapid, strong and, reversible intracellular vacuolization in multiple types of cancer cells. **(A)** Morphological changes of A549 and PANC-1 cells treated with R491 at different concentrations of for 72 h. **(B)** Morphological changes of A549 and PANC-1 cells treated with R491 at 1 µM for different lengths of time. **(C)** Morphology of A549 cells treated with R491 at 1 µM for 24 h, and at different time after the removal of R491 from the culture. **(D)** Morphological changes of U87, HUH7, and AGS cells treated with 1 µM R491 for 72 h. Scale bars: 50 µm.

To evaluate the effect of the vimentin-binding compound R491, human cancer cells from non-small cell lung cancer A549, pancreatic cancer PANC-1, glioma U87, hepatoma HUH7, and gastric cancer AGS were cultured in the presence of the solvent DMSO or the compound R491 at different concentrations for different periods of time, morphological changes were examined under microscope. Intracellular vacuoles became visible within 2 h after the exposure of cells to the compound at a concentration of 1 μM (Figures 1A,B). This vacuolization showed a clear dose and time dependency (Figures 1A,B). The vacuoles accumulated overtime inside the cells when the compound was present but diminished rapidly after the removal of the compound from the culture. The intracellular vacuoles remarkably reduced in number within 2 h and disappeared within 12 h after the compound removal (Figure 1C). All the cancer cell lines tested showed the similar phenotype responding to the compound treatment (Figure 1D).

### The Vimentin-Binding Compound R491 had No Effects on Cancer Cell Growth or Levels of Major Oncoproteins

**FIGURE 2:**
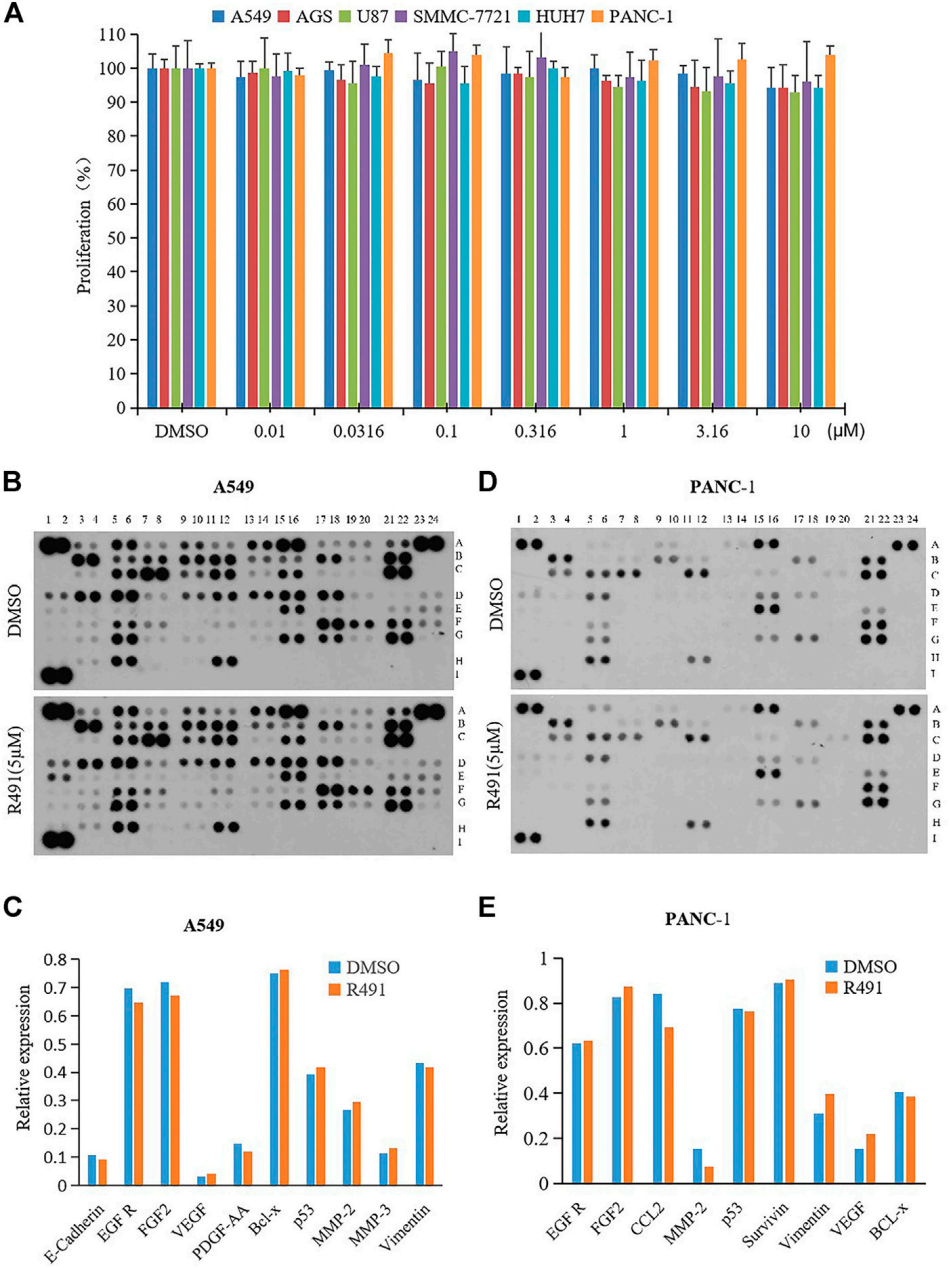
Lack of effects on cancer cell growth and on levels of vimentin and major oncoproteins. **(A)** R491 did not inhibit proliferation of cancer cells A549, AGS, U87, SMMC-7221, HUH7, and PANC-1. **(B, D)** Semi-quantification of oncoproteins in **(B)** A549 or **(D)** PANC-1 cells by the Human XL Oncology. The lysates were prepared from A549 or PANC-1 cells treated with either DMSO or R491 at 5 µM for 48 h. See Supplementary Figure S1 and Supplementary Table S2 for coordinates detail. **(C, E)** The graph summarizes the relative signal intensity of indicated proteins in **(C)** A549 or **(E)** PANC-1 cells.

To examine whether the phenotypic alteration induced by the vimentin-binding compound R491 would result in inhibition on cancer cell growth, various types of human cancer cells, including A549, AGS, U87, SMMC-7721, HUH7, and PANC-1 were treated with the compound R491 at concentrations up to 10μM, no growth inhibition was observed in any of these cells at any concentrations (Figure 2A).

To understand whether the phenotypic alteration induced by the vimentin-binding compound in cancer cells would relate to any oncogenic signaling pathways, a protein chip study was performed. Using the protein array that consists of 84 selected human cancer-related proteins (Supplementary Figure S1 and Supplementary Table S2), the compound R491 showed minimal or no effects on the protein levels of vimentin and other signaling molecules related to cell proliferation in both A549 (Figures 2B,C) and PANC-1 cells (Figures 2D,E).

### Intracellular Vacuole Accumulation Induced by the R491 Treatment Represented a Disturbed Exosomal Pathway

**FIGURE 3:**
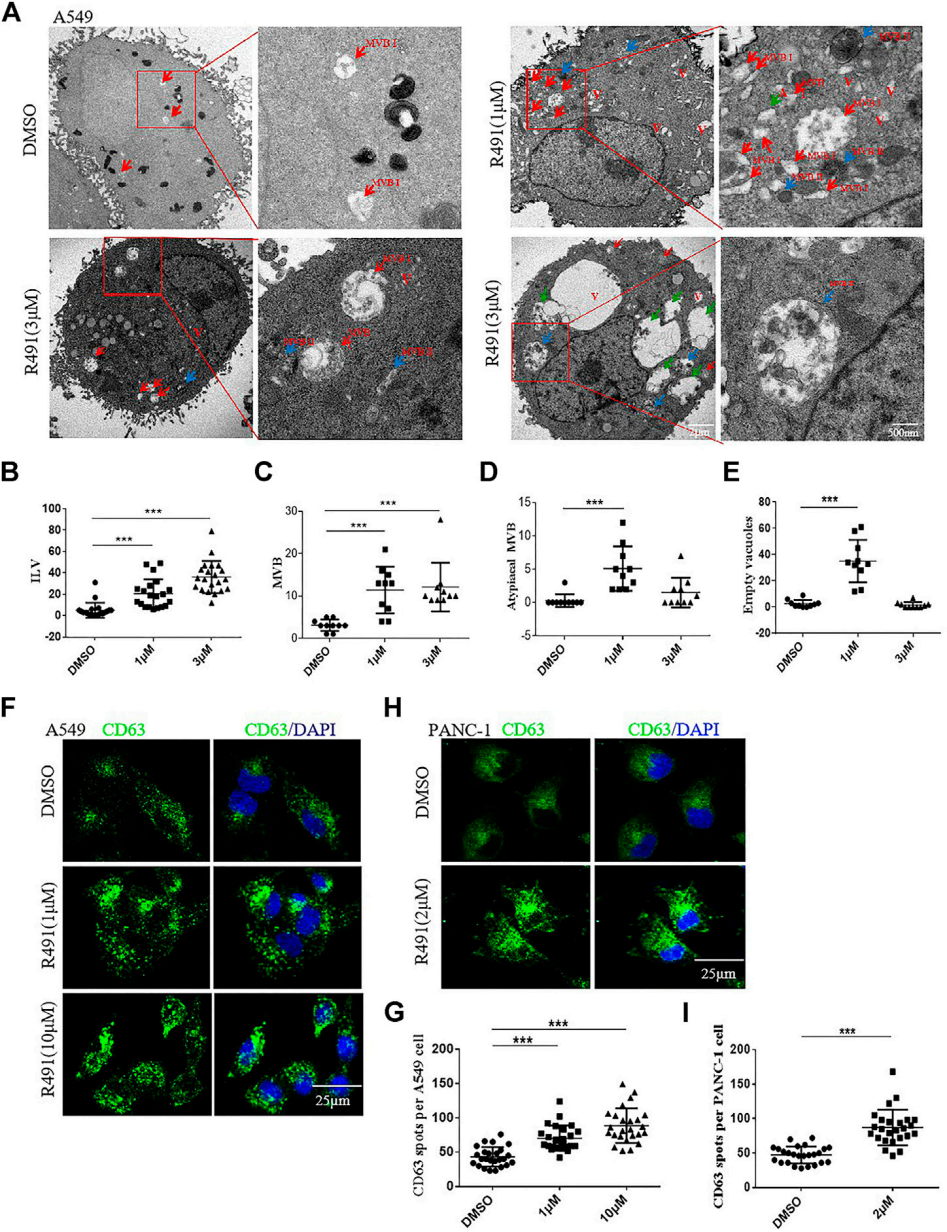
Analysis of the intracellular vacuole accumulation induced by R491 revealed a disturbed exosomal pathway. **(A)** Representative transmission electron micrographs of A549 cells treated with DMSO or R491 (1 and 3 µM), showing morphologies of MVB I (red arrows), MVB II (blue arrows), atypical MVB II (green arrows) and empty vacuoles (V). Scale bars: 2 μm; 500 nm. **(B–E)** Quantification of organelles present in the cells treated with DMSO and R491(1 and 3 µM). Number of ILVs **(B)** in each MVB (n = 20); numbers of MVBs **(C)**, atypical MVBs **(D)** and empty vacuoles (V) **(E)** per cell (n = 10) were expressed as mean ± SEM. Statistical significance: ***p < 0.001 as determined by Mann-Whitney test. **(F, H)** Confocal microscopy analysis of the endosome markers CD63 (green) in the A549 **(F)** and the PANC-1 **(H)** cells treated with DMSO and R491 (1 and 10 µM). Scale bar: 25 µm. **(G, I)** Quantification of the number of CD63^+^ particles per A549 **(G)** and PANC-1 **(I)** cell. The dot plot represents the number of CD63^+^ from individual cells (n = 25). The data are expressed as mean ± SEM (n = 25). ***p < 0.001 as determined by two-tailed Student’s t-test.

To characterize the nature of the accumulated vacuoles, we used transmission electron microscopy to visualize the structure and content of these vacuoles in the R491-treated A549 cells. Heterogeneous types of vacuoles were identified and classified based on their morphology, which included multivesicular bodies-I (MVB I) containing numerous intraluminal vesicles (ILVs), MVB II with degrading ILVs, atypical MVB II with contents apparently degraded, and transparent (or empty) vesicles (Figure 3A). The MVBs are normally destined either for degradation by fusing with lysosomes, or for extracellular release of their contents by fusing with the cell membrane. The different types of vesicles in treated cells appeared to be at different stages of the degradation process. Both MVBs and ILVs were significantly and dose-dependently increased after the R491 treatment (Figures 3B,C). Atypical MVB II and empty vesicles significantly increased after the treatment of cells with R491 at 1 µM, but not at the higher concentration of 3 µM, which may suggest dynamic responses to R491 by different types of vesicles (Figures 3D,E). To further investigate the subcellular distribution of MVBs, immunofluorescence imaging was performed using CD63 as a marker of MVBs. The compound induced a densely clustered cytoplasmic localization of CD63, as shown by the increased size and number of CD63^+^ spots in R491 treated A549 cells **(** Figures 3F,G) as well as in PANC-1 cells (Figures 3H,I).

### The Vimentin-Binding Compound R491 Inhibited the Exosome Secretion but didn’t Reduce the Exosome Generation

**FIGURE 4:**
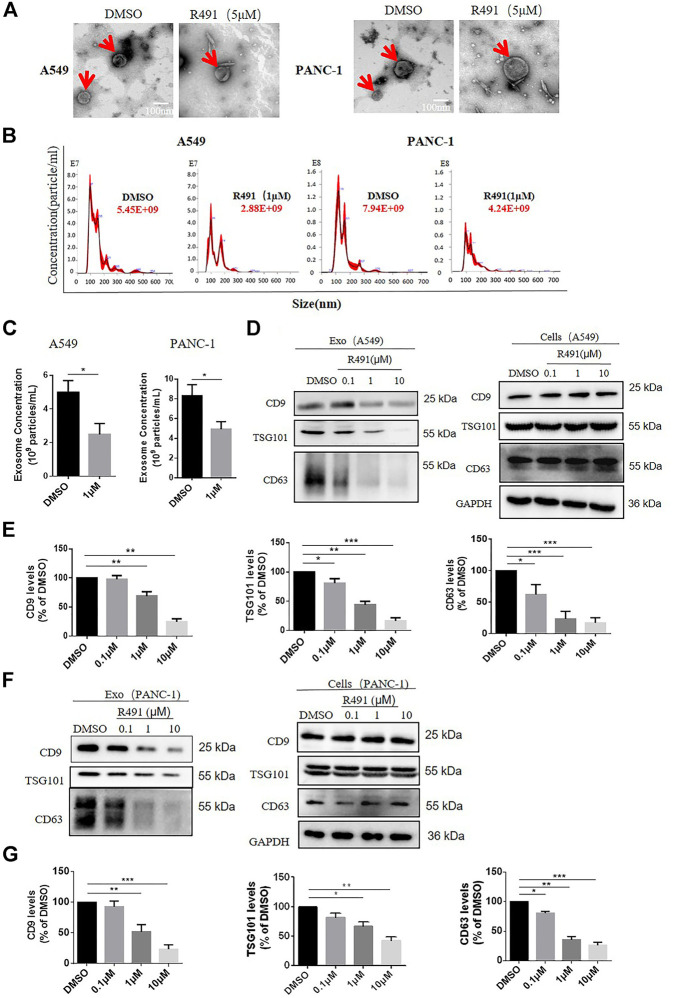
The vimentin-binding compound R491 inhibited exosomes secretion rather than exosomes generation. **(A)** Representative transmission electron micrographs of exosomes (red arrows) purified from A549 and PANC-1 cells treated with DMSO or R491 at 5 µM scale bars: 100 nm. **(B)** Nanoparticle tracking analysis (NTA) of small extracellular vesicles (sEVs) released from A549 and PANC-1 cells treated with DMSO or R491 at 1 µM sEVs were isolated by serial ultracentrifugation from cell culture supernatants of same number of cells. **(C)** NTA quantification of three independent experiments. **(D)** Western blot analysis of A549 cells treated with DMSO or with R491 at 0.1, 1, and 10 µM for 48 h. Extracts from cells and sEVs (Exo) were blotted for the exosomal markers CD9, CD63, and TSG101. **(E)** Quantification of exosomal proteins extracted from sEVs of A549 cells in three independent experiments. **(F)** Western blot analysis of PANC-1 cells treated with DMSO or with R491 at 0.1, 1 and 10 µM for 48 h. Extracts from cells and sEVs (Exo) were blotted for the exosomal markers CD9, CD63 and TSG101. **(G)** Quantification of exosomal proteins extracted from sEVs of PANC-1 cells in three independent experiments.

To further dissect the effects of R491 on exosomes, we purified and quantified the exosomes in the media of cultured cells. Using the classic exosome purification method through three rounds of centrifugation, we obtained the exosomes with a morphology of typical disk-like structures mostly in diameters of 50–150 nm (Figure 4A). Nano-tracking analysis showed a significant reduction of exosomes in the culture supernatant of A549 cells as well as PANC-1 cells that were treated with the compound R491 (Figures 4B,C). Western blot also confirmed that the compound R491 treatment resulted in reduced amount of exosome markers in the exosome preparations (Figures 4D–G). In contrary, intracellular exosome levels were not reduced by the compound R491 treatment (Figures 4D,F), suggesting the compound R491 blocks exosome release from the cells rather than exosome generation inside the cells.

### R491 Inhibited Exosome Secretion by Targeting Vimentin

**FIGURE 5:**
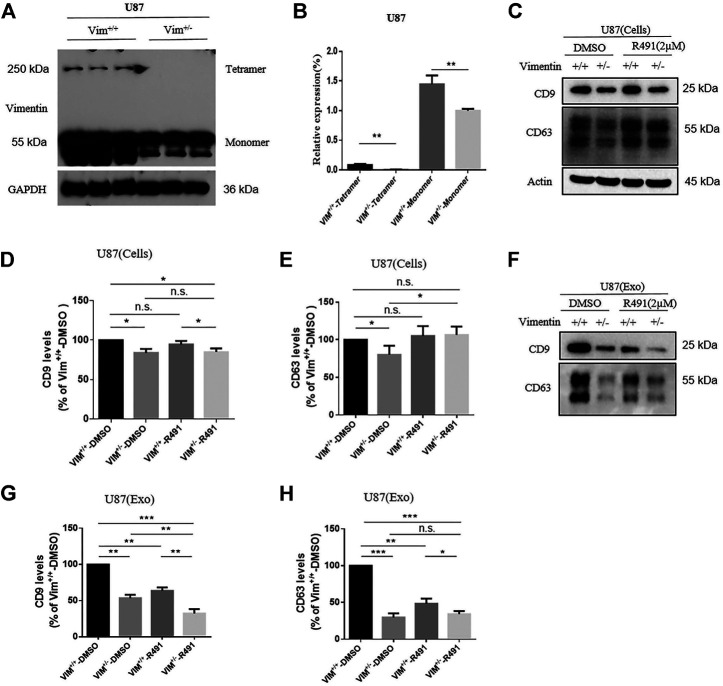
Vimentin regulates exosome formation and release while the vimentin-binding compound R491 inhibits exosome release only. **(A)** A representative western blot analysis of vimentin tetramer and vimentin monomer from cell lysates of U87 Vim^+/+^ and U87 Vim^+/-^ cells. **(B)** Quantification of the protein levels of vimentin from **(A)** in three independent experiments. **(C)** A representative western blot analysis of cell lysates from equal numbers of U87 Vim^+/+^ and U87 Vim^+/-^ cells after being treated with DMSO or with 2 µM of R491 for 72 h. Cell lysates (Cells) were blotted for the exosomal markers CD9 and CD63, and *β*-actin as a loading control. **(D–E)** Quantification of the protein levels of exosomal markers CD9 and CD63 from **(C)** in three independent experiments. **(F)** A representative western blot analysis of the lysates of extracellular vesicles purified from the cultures with equal numbers of U87 Vim^+/+^ and U87 Vim^+/-^ cells after being treated with DMSO or with 2 µM of R491 for 72 h. Extracellular vesicles (Exo) were extracted and blotted for the exosomal markers CD9 and CD63. **(G–H)** Quantification of exosomal markers CD9 and CD63 from **(F)** in three independent experiments. The data are expressed as mean ± SEM (n = 3). *p < 0.05, **p < 0.01 and ***p < 0.001 as determined by two-tailed Student’s t-test.

To determine whether the exosome release blockade by R491 is the direct result of its vimentin targeting, we sought to knock out vimentin gene from human glioma U87 cells using the CRISPR-cas9 system. We were not able to obtain Vim^−/-^ clones, but only Vim^+/−^ clones. The absence of viable Vim^−/−^ clones may suggest a critical role of vimentin in this particular cancer type. Western blot showed that, compared with U87 Vim^+/+^ cells, U87 Vim^+/−^ cells had lower amounts of intracellular vimentin protein (Figures 5A,B), and accordingly, lower amounts of intracellular exosomes (Figures 5C–E), suggesting that vimentin promotes exosome formation inside the cells. The R491 treatment did not reduce the amount of intracellular exosomal marker CD9 in either U87 Vim^+/+^ cells or in U87 Vim^+/−^ cells (Figures 5C,D), did not reduce the amounts of intracellular exosomal makers CD63 in U87 Vim^+/+^ cells, but in contrary, increased CD63 in U87 Vim^+/−^ cells (Figures 5C,E). These results indicate that R491 treatment, unlike vimentin knockdown, did not reduce the amounts of intracellular exosomes (or ILVs). Western blots (Figure 5F) and the quantification (Figures 5G,H) of the exosomes released from U87 Vim^+/+^ and U87 Vim^+/−^ cells showed that both vimentin knockdown and the R491 treatment reduced the amounts of exosomal markers CD9 and CD63 (Figures 5F–H), and the R491 treatment resulted in a further reduction of CD9 in the U87 Vim^+/−^ cells (Figures 5F,G). These results indicate that the vimentin-binding compound R491 mainly blocked the release of exosomes from the cells.

### The Vimentin-Binding Compound R491 Significantly Inhibited Cancer Cell Migration and Invasion in Intro and Reduced Exosomes in the Blood in Mice

**FIGURE 6:**
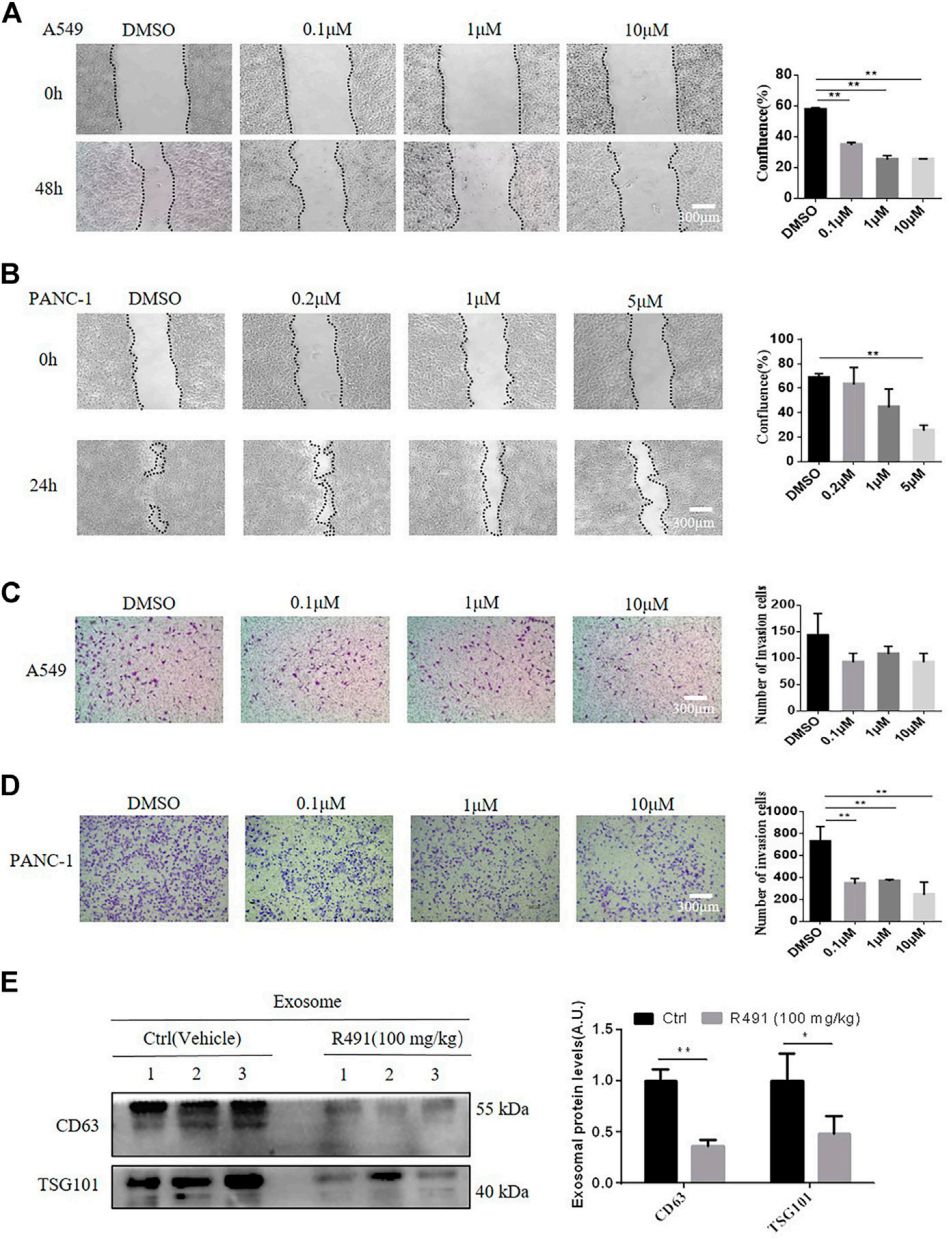
The vimentin-binding compound R491 significantly inhibits cancer cell migration and invasion. **(A, B)** The wound assay, showing the migration of the A549 and PANC-1 cells before and after treated with R491 at different concentrations for 48 h **(A)** or 24 h **(B)** duration (left), and the quantification of confluence (right). Scale bars: 300 µm. **(C, D)** Transwell invasion assay, showing the migration of **(C)** A549 or **(D)** PANC-1 cells treated with DMSO or with R491 at different concentrations for 24 h (left), and quantification of the migrated cells (right). **(E)** Western blot analysis of exosomal marker CD63 and TSG101 from sEVs purified by sucrose density gradient centrifugation from plasma of mice after the administration of R491 (100 mg/kg, i.g., once daily, n = 3) or vehicle (n = 3) for 14 days. Quantification of exosomal proteins in three independent Western blots. Scale bars: 300 µm. The data are expressed as mean ± SEM (n = 3). *p < 0.05, **p < 0.01 as determined by two-tailed Student’s t-test.

As it’s well known that vimentin promotes cell migration and cancer exosomes increase cell mobility (Ivaska et al., 2007; Hoshino et al., 2015), we investigated whether the vimentin-binding compound would have impacts on cancer cell migration and invasion. The wound-healing assay showed the rate of gap size closure over time significantly decreased in response to the treatment with R491 in a dose-dependent manner in both A549 cells (Figure 6A) and PANC-1 cells (Figure 6B). The transwell assay showed that R491 impeded the ability of cells to migrate for both cancer types, more significantly for PANC-1 cells (Figure 6D) than for A549 cells (Figure 6C). As PANC-1 cells migrated faster than A549 cells, it appeared that the vimentin-binding compound R491 had more impacts on more aggressive cancer cells.

To confirm the activity of R491 *in vivo*, we administered the compound to mice via oral gavage daily for 14 days. sEVs were purified from the mouse plasma and quantified by Western blot for exosome markers. Both CD63 and TSG101 were significantly decreased in the sEV isolated from the R491-treated mice than that from vehicle -treated mice (Figure 6E).

## Discussion

Vimentin is a potential target for cancer treatment (Strouhalova et al., 2020) since it is critically involved in the cellular processes, such as epithelial-to-mesenchymal transition (EMT) (Liu et al., 2015), cell migration and invasion (Sharma et al., 2019), that are the common features of all solid tumors, not limited to a particular tumor type. However, the approaches of targeting vimentin have yielded little success in treating tumors, probably because all the previous attempts focused on finding vimentin inhibitors that can directly kill cancer cells but ignored the fact that the depletion of vimentin itself does not result in the death of normal cells (Colucci-Guyon et al., 1994) or cancer cells, except under extreme non-physiological conditions or special circumstances where synthetic lethal may occur (Bollong et al., 2017). The roles of vimentin in inter-cellular communication have been largely unexplored, however, interaction between cancer and normal cells are the keys for the establishment of tumor microenvironment (TME) and tumor metastasis where exosomes are the critical mediators. The roles of exosomes in the cancer immune surveillance make the exosome inhibition a very attractive anticancer approach (Kurywchak et al., 2018).

Our previous proteomic profiling study identified vimentin as the specific target of a new type of vimentin-binding compound (Zhang et al., 2021). We wanted to understand the biological consequences of the interaction between this type of compound and vimentin. Unlike withaferin A and FiVe1 (Bargagna-Mohan et al., 2007; Bollong et al., 2017), which are known vimentin inhibitors that degrade vimentin, our vimentin-binding compound R491 does not reduce the quantity of vimentin protein nor inhibit cancer cell growth (Figure 2), but instead alters the physical property of vimentin filaments (unpublished data) and compromises the mobility of cancer cells. Vimentin, as a canonical marker of epithelial to mesenchymal transition (EMT), is strongly associated with cancer invasive phenotype (Ivaska et al., 2007; Gonzalez and Medici, 2014). The vimentin-binding compound R491 may be effective against cancer invasion. The fast-acting, reversible and non-cytotoxic nature of the target binding makes R491 a desirable drug candidate worth further investigation.

The vimentin-binding compound R491 induced intracellular vacuolization in various types of cancer cells (Figure 1). This phenotype is not surprising because vimentin has important roles in membrane trafficking, providing tracks for vesicle transport (Styers et al., 2006). Characterization of these vacuolized cells indicated that the intracellular vacuoles were enlarged exosome-containing multivesicular bodies at different degradation stages (Figure 3). Quantification of exosomes revealed that the vimentin-binding compound R491 reduced the extracellular but not intracellular exosomal markers (Figures 4D–F), suggesting a blockade on exosome release rather than on exosome generation. To ascertain that the exosome release blockade by R491 were the direct result of its vimentin targeting, we used the CRISPR/case9 system to knockout one allele of vimentin gene from U87 glioma cells. Indeed, U87 Vim^+/−^ cells that had half amount of vimentin protein, released only about 50% of the exosomes released from U87 Vim^+/+^. The vimentin-binding compound R491 reduced exosome release from U87 Vim^+/+^ and further from U87 Vim^+/−^ (Figures 5E,F). These results may indicate that vimentin favors exosome release and the vimentin-binding compound R491 blocks exosome release through its vimentin targeting. Interestingly, the effects of the vimentin knockdown and the vimentin-binding compound R491 on intracellular exosomes (ILVs) were different. The vimentin knockdown reduced intracellular exosomal makers as it did to released exosomes, while the vimentin-binding compound R491 was unable to reduce intracellular exosomal markers but only the amounts of released exosomes (Figure 5). This difference may be explained by the fact that the vimentin knockdown reduced intracellular vimentin protein levels, while the vimentin-binding compound R491 did not (Figures 2B–E). Therefore, the function of vimentin involved in exosome release may be uncoupled with its function in exosome biogenesis or other essential processes, which may provide a possibility of targeting vimentin specifically for blocking exosome release without interfering other normal cellular processes or functions. Indeed, in the mice received oral treatment of R491 daily for 14 days, no abnormalities were observed, while the amount of exosome in the plasma was significantly reduced (Figure 6E).

As exosomes play important roles in both normal biological processes and the pathogenesis of a variety of diseases, modulating exosomes represents an attractive therapeutic approach, especially for cancer (van Niel et al., 2018; Pegtel and Gould, 2019; Kalluri and LeBleu, 2020). Cancer cells release a large number of exosomes. Cancer exosomes are increasingly recognized as a central mediator in cancer formation, progression, metastasis, and drug resistance. Cancer exosomes transfer cancer-specific materials to non-cancer cells to form tumor microenvironments (TME) that support the survival and proliferation of cancer stem cells (CSCs) and other tumor cells. Cancer exosomes help cancer grow with angiogenesis, gain migratory and invasive capacity, and evade immune surveillance. Cancer exosomes carrying PD-L1 can mediate immunosuppression locally or systemically, resisting immune checkpoint therapy (Chen et al., 2018). Inhibiting exosomal PD-L1 can induce systemic anti-tumor immunity with lasting anti-tumor activity (Poggio et al., 2019). Therefore, targeting vimentin to reduce exosome release appears to be a promising approach to developing anticancer therapy.

In conclusion, our results demonstrate that vimentin promotes the biogenesis and the release of cancer exosomes, and that a reversible vimentin-binding compound can block cancer exosome release and inhibit cancer cell migration and invasion without cytotoxic effects. Our study provides a potential therapeutic strategy for cancer by targeting vimentin to block cancer exosome release.

## Data Availability

The raw data supporting the conclusions of this article will be made available by the authors, without undue reservation.
