# Supplementary material for: A Small Vimentin-Binding Molecule Blocks Cancer Exosome Release and Reduces Cancer Cell Mobility
Source: Front Pharmacol. 2021 Jul 8;12:627394. doi: 10.3389/fphar.2021.627394 (PMC8297618; doi:10.3389/fphar.2021.627394)
Supplement: Supplementary file 1 [file Table1.pdf]

Supplementary Table S1 │ Certificate of Analysis

**Product Code**	**Bellen 00014511(LD-R491)**		
Batch No.	180101	Batch Size	13.3kg
Check Department	——	Check Date	2018.11.25
Manufacturing Date	2018.07.31	Retest Date	2019.11.24

**Test Item**	**Specification**		**Result**
Identification by IR	Consistent with the reference spectrum		Consistent with the reference spectrum
Identification by UV	Consistent with the reference spectrum		Consistent with the reference spectrum
Identification by DSC	Consistent with the reference spectrum		Consistent with the reference spectrum
Identification by XRPD	Consistent with the reference spectrum		Consistent with the reference spectrum
Identification by HNMR	Consistent with its structure		Consistent with its structure
Identification by TGA	Consistent with the reference spectrum		Consistent with the reference spectrum
Identification by LC/MS	Consistent with its structure		Consistent with its structure
Residue on ignition（% m/m）	NMT 1.0		0.25
Water content（K.F., % m/m）	NMT 1.0		0.08
Residual solvents (GC-HS, % m/m)	Tetrahydrofuran	NMT 0.8	0.3
	Dichloromethane	NMT 0.06	Not Detected
	Dimethyl sulfoxide	NMT 0.5	0.1
	Ethanol	NMT 0.5	0.02
Residual solvents (IC, % m/m)	Acetic acid	NMT 0.5	Not Detected
Particle size（μm）	D10、D50、D90：Report result		D10 = 2.99<br>D50 = 205<br>D90 = 1190

Assay (HPLC, % w/w)	Assay<br>NMT 96.0		99.0
Purity (HPLC, % area)	Purity	NMT 98.0	99.5
	Maximum of single impurity	NMT 0.5	0.15
Conclusion	The product is tested to be qualified		

Analyst：signed Reviewer：signed Approver signed

Date： 01/20/2019 Date： 01/20/2019 Date：01/20/2019

By： Porton Pharma Solutions, Ltd.
1 Fine Chemical Zone, Chongqing Chem. Ind Park, Changshou, Chongqing, 401221, China

TEL: +86 (0)23 86083200#5347, FAX: +86 (0)23 65936901
