# Supplementary material for: A Small Vimentin-Binding Molecule Blocks Cancer Exosome Release and Reduces Cancer Cell Mobility
Source: Front Pharmacol. 2021 Jul 8;12:627394. doi: 10.3389/fphar.2021.627394 (PMC8297618; doi:10.3389/fphar.2021.627394)
Supplement: Supplementary file 2 [file Table2.pdf]

Supplementary Table S2 │ Microarray protein IDs.

Coordinate	Analyte/Control	Entrez Gene ID	Alternate Nomenclature
A1, A2	Reference Spots	N/A	_____
A3, A4	α-Fetoprotein	174	AFP, DSCAM2
A5, A6	Amphiregulin	374	AREG
A7, A8	Angiopoietin-1	284	ANGPT1
A9, A10	Angiopoietin-like [illegible]	51129	ANGPTL4
A11, A12	ENPP-2/Autotaxin	5168	ATX, Lysophosphatidic Acid, NPP2, PDNP2
A13, A14	Axl	558	Ark, Ufo
A15, A16	BCL-x	598	BCL2L1
A17, A18	CA125/MUC16	94025	MUC16
A19, A20	E-Cadherin	999	Arc-1, CAD1, Cadherin-1, CD324, CDH1, Cell-CAM 120/80, ECAD, L-CAM
A21, A22	VE-Cadherin	1003	Cadherin-5, CD144, CDH5
A23, A24	Reference Spots	N/A	_____
B3, B4	CapG	822	AFCP
B5, B6	Carbonic Anhydrase IX	768	CA9, G250, MN, RCC
B7, B8	Cathepsin B	1508	CTSB
B9, B10	Cathepsin D	1509	CTSD
B11, B12	Cathepsin S	1520	CTSS
B13, B14	CEACAM-5	1048	CD66e, CEA
B15, B16	Decorin	1634	DCN, DSPG2, PG-II, PSG2, SLRR1B
B17, B18	Dkk-1	22943	Dickkopf-1
B19, B20	DLL1	28514	Delta 1
B21, B22	EGF R/ErbB1	1956	ErbB, ErbB1, HER-1
C3, C4	Endoglin/CD105	2022	CD105, ENG
C5, C6	Endostatin	80781	COL18A1

C7, C8	Enolase 2	2026	ENO2; γ-Enolase; NSE
C9, C10	eNOS	4846	NOS3
C11, C12	EpCAM/TROP1	4072	17-1A, CD326, GA733-2, gp40, KS1/4,
C13, C14	ERα/NR3A1	2099	ESR1, NR3A1
C15, C16	ErbB2	2064	CD340, HER2, Neu Oncogene, NGL, TKR1
C17, C18	ErbB3/Her3	2065	HER3
C19, C20	ErbB4	2066	HER4
C21, C22	FGF basic	2247	FGF2, FGF-2, FGF2AS, GFG1, HBGH-2, NUDT6, Prostatropin
D1, D2	FoxC2	2303	Fkh14, LD, MFH1
D3, D4	FoxO1/FKHR	2308	FKH1, FKHR
D5, D6	Galectin-3	3958	AGE-R3, CBP35, GAL3, L29, LGALS3, Mac-2
D7, D8	GM-CSF	1437	CSF2
D9, D10	CG α/β (HCG)	1081(α)/1082(β)	CGB, CGB3, Choriogonadotropin
D11, D12	HGF R/c-Met	4233	MET
D13, D14	HIF-1α	3091	HIF1A
D15, D16	HNF-3β	3170	FoxA2
D17, D18	HO-1/HMOX1	3162	HSP32
D19, D20	ICAM-1/CD54	3383	———
D21, D22	IL-2 Rα	3559	CD25, IL2RA
D23, D24	IL-6	3569	BSF-2, IFN-β2, MGI-2A
E1, E2	CXCL8/IL-8	3576	GCP1, IL8, LAI, MDNCF, NAP1, NCF, TCF,TSG1
E3, E4	IL-18 BPa	10068	IL18BP
E5, E6	Kallikrein 3/PSA	354	KLK3
E7, E8	Kallikrein 5	25818	KLK5, KLK-L2, SCTE
E9, E10	Kallikrien 6	5653	KLK6, Neurosin, Protease M,
E11, E12	Leptin	3952	LEP, OB
E13, E14	Lumican	4060	LDC, LUM, SLRR2D

E15, E16	CCL2/MCP-1	6347	MCAF
E17, E18	CCL8/MCP-2	6355	_____
E19, E20	CCL7/MCP-3	6354	MARC
E21, E22	M-CSF	1435	CSF1, CSF-1
E23, E24	Mesothelin	10232	CAK1, MPF, MSLN, SMR
F1, F2	CCL3/MIP-1α	6348/6351	LD78a; MIP-1 alpha
F3, F4	CCL20/MIP-3α	6364	exodus-1; LARC; MIP-3 alpha
F5, F6	MMP-2	4313	Gelatinase A
F7, F8	MMP-3	4314	Stromelysin-1
F9, F10	MMP-9	4318	CLG4B, Gelatinase B, GELB
F11, F12	MSP/MST1	4485	HGFL, MST1, SF2
F13, F14	MUC-1	4582	CD227, Episialin, H23AG, KL-6, Mucin-1, PEM, PEMT
F15, F16	Nectin-4	81607	LNIR, PRR4, PVRL4
F17, F18	Osteopontin (OPN)	6696	Eta-1, Spp1
F19, F20	p27/Kip1	1027	CDKN1B
F21, F22	p53	7157	BCC7, LFS1, TP53, TRP53
F23, F24	PDGF-AA	5154	_____
G1, G2	CD31/PECAM-1	5175	PECAM1
G3, G4	Progesterone R/NR3C3	5241	_____
G5, G6	Progranulin	2896	Acrogranin, GEP, GP88, GRN, PCDGF, PEPI, PGRN, Proepithelin
G7, G8	Prolactin	5617	PRL
G9, G10	Prostasin/Prss8	5652	_____
G11, G12	E-[illegible]	6401	ELAM1, LECAM2, SELE
G13, G14	Serpin B5/Maspin	5268	PI5
G15, G16	Serpin E1/PAI-1	5054	Nexin, PLANH1
G17, G18	Snail	6615	SLUGH2, SNAH, SNAI1
G19, G20	SPARC	6678	BM-40, Osteonectin
G21, G22	Survivin	332	API4, BIRC5

G23, G24	Tenascin C	3371	Cytotactin, HXB, Tenascin J1, TNC
H1, H2	Thrombospondin-1	7057	THBS1, TSP-1
H3, H4	Tie-2	7010	_____
H5, H6	u-Plasminogen Activator/Urokinase	5328	PLAU, uPA
H7, H8	VCAM-1/CD106	7412	_____
H9, H10	VEGF	7422	VAS, Vasculotropin, VEGFA, VPF
H11, H12	Vimentin	7431	VIM
I1, I2	Reference Spots	N/A	_____
I23, I24	Negative Control	N/A	_____
