# Supplementary figures and images for: A Small Vimentin-Binding Molecule Blocks Cancer Exosome Release and Reduces Cancer Cell Mobility
Source: Front Pharmacol. 2021 Jul 8;12:627394. doi: 10.3389/fphar.2021.627394 (PMC8297618; doi:10.3389/fphar.2021.627394)

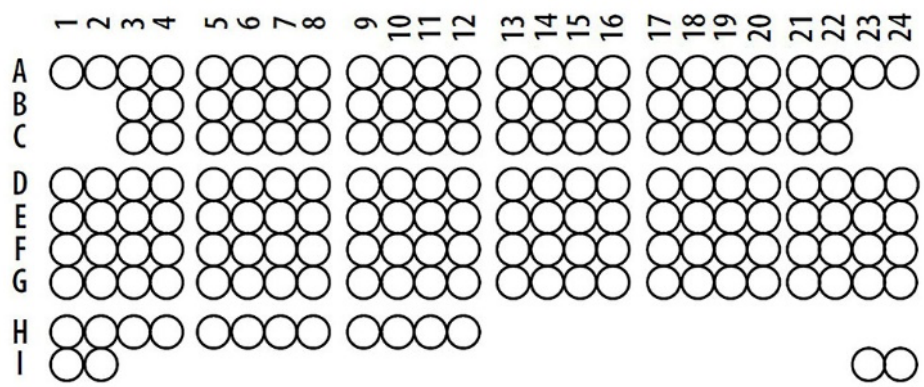

**Supplementary Figure S1** | Human XL Oncology Array Coordinates.

Supplement: Supplementary file 3 [file Image1.pdf]
